# High frequencies of nonviral colds and respiratory bacteria colonization among children in rural Western Uganda

**DOI:** 10.3389/fped.2024.1379131

**Published:** 2024-05-02

**Authors:** Taylor E. Weary, Tressa Pappas, Patrick Tusiime, Shamilah Tuhaise, Elizabeth Ross, James E. Gern, Tony L. Goldberg

**Affiliations:** ^1^ Department of Pathobiological Sciences, University of Wisconsin School of Veterinary Medicine, Madison, WI, United States; ^2^ Department of Pediatrics, University of Wisconsin School of Medicine and Public Health, Madison, WI, United States; ^3^ The Kasiisi Project, Fort Portal, Uganda

**Keywords:** respiratory disease, pediatrics, Uganda, COVID-19, asthma, COPD, air pollution

## Abstract

**Introduction:**

Respiratory illness is the most common childhood disease globally, especially in developing countries. Previous studies have detected viruses in approximately 70-80% of respiratory illnesses.

**Methods:**

In a prospective cohort study of 234 young children (ages 3-11 years) and 30 adults (ages 22-51 years) in rural Western Uganda sampled monthly from May 2019 to August 2021, only 24.2% of nasopharyngeal swabs collected during symptomatic disease had viruses detectable by multiplex PCR diagnostics and metagenomic sequencing. In the remaining 75.8% of swabs from symptomatic participants, we measured detection rates of respiratory bacteria *Haemophilus influenzae, Moraxella catarrhalis,* and *Streptococcus pneumoniae* by quantitative PCR.

**Results:**

100% of children tested positive for at least one bacterial species. Detection rates were 87.2%, 96.8%, and 77.6% in children and 10.0%, 36.7%, and 13.3% for adults for *H. influenzae, M. catarrhalis,* and *S. pneumoniae*, respectively. In children, 20.8% and 70.4% were coinfected with two and three pathogens, respectively, and in adults 6.7% were coinfected with three pathogens but none were coinfected with two. Detection of any of the three pathogens was not associated with season or respiratory symptoms severity, although parsing detection status by symptoms was challenged by children experiencing symptoms in 80.3% of monthly samplings, whereas adults only reported symptoms 26.6% of the time. Pathobiont colonization in children in Western Uganda was significantly more frequent than in children living in high-income countries, including in a study of age-matched US children that utilized identical diagnostic methods. Detection rates were, however, comparable to rates in children living in other Sub-Saharan African countries.

**Discussion:**

Overall, our results demonstrate that nonviral colds contribute significantly to respiratory disease burden among children in rural Uganda and that high rates of respiratory pathobiont colonization may play a role. These conclusions have implications for respiratory health interventions in the area, such as increasing childhood immunization rates and decreasing air pollutant exposure.

## 1 Introduction

Respiratory illness is the most common childhood disease globally, especially in low- and middle-income countries (LMICs) (1). Seminal studies detected viruses in 70%–80% of respiratory illnesses, but these studies sampled populations in affluent locations, primarily the US (2, 3) or European countries (4). However, these studies lack generalizability to children in LMICs, where the burden of bacterial disease is much higher and uptake of conjugate vaccines for respiratory pathogens *Streptococcus pneumoniae* (pneumococcus, PCV) and *Haemophilus influenzae* type b (Hib) is much lower (5). There are currently few comparable data from Sub-Saharan Africa, which vary by location and methodology. In a study of Ugandan children under five years admitted to a hospital for febrile respiratory illness, 80.0% of nasopharyngeal swabs tested positive for a virus by metagenomics (6), whereas only 48.8% and 45.7% tested PCR-positive among children of the same age admitted to intensive care units in Mozambique (7) and South Africa (8), respectively. Because most people do not seek medical care for relatively mild respiratory illnesses, particularly where health care access is limited, such hospital-based studies may not accurately capture prevalence rates of viral and nonviral colds. We sought to characterize respiratory illness etiologies in rural Western Uganda through a community-based longitudinal study.

We conducted a prospective cohort study of young children and adults in rural communities in Kabarole District, Western Uganda (9). Each month from May 2019 to August 2021, we collected paired respiratory symptoms surveys and nasopharyngeal swabs by visiting participants at their schools, homes, and workplaces. We first examined whether respiratory symptoms were caused by common respiratory pathogens using a multiplex PCR panel that detects 17 viruses and three bacteria. We measured frequencies of both viral and nonviral respiratory disease and compared them to elsewhere (2–4, 10).

We then measured CXC chemokine ligand 10 (CXCL10) in nasopharyngeal swabs. Induced by interferon-γ, CXCL10 plays an important role in both innate and adaptive antiviral immune responses by inducing chemotaxis of NK cells, macrophages, dendritic cells, and T lymphocytes to sites of infection (11) and polarizing Th1 cells (12). Elevated CXCL10 levels can indicate active viral infection (13–15). We therefore examined individuals with high CXCL10 levels using metagenomics to investigate rare or novel viruses unrepresented on our multiplex PCR panel.

Next, we measured carriage of respiratory pathobionts (pathogenic bacteria that first colonize the upper respiratory tract as commensals) *Haemophilus influenzae, Moraxella catarrhalis,* and *Streptococcus pneumoniae*. These pathobionts often cause acute otitis media, sinusitis, and pneumonia in children (16) as well as exacerbations of chronic obstructive pulmonary disease in adults (17, 18). Pathogenic microbiota overgrowth generally follows acute insult from either respiratory viral infections or air pollutants. Detection rates and densities increase after respiratory virus infection by inducing cellular receptors used by bacteria for adhesion (19, 20) and disrupting epithelial barrier function (21). Bacterial components can activate Toll-like receptors, prompting the release of large amounts of inflammatory cytokines, resulting in increased risk of wheezing illnesses and asthma exacerbations (10, 22, 23). Additionally, environmental and household air pollution can increase levels of these pathobionts, especially in many parts of Sub-Saharan Africa (e.g., South Africa (24), Ghana (25), and Malawi (26)) where annual fine particulate matter (PM_2.5_) concentrations exceed WHO standards by as much as 10-fold (27). To contrast, we compared respiratory pathobiont prevalence among children in this study with prevalence among age-matched, suburban children from a US cohort study that utilized the same diagnostic methods (10), allowing for direct comparison of these two populations.

Fortuitously, the study occurred before, during, and after the emergence COVID-19 when public health measures (e.g., closure of schools and businesses, prohibition of travel internationally or between districts in-country, mandatory mask-wearing, etc.) were instituted, allowing us to capture the changing dynamics of respiratory disease during a time of intense public health measures with the goal of reducing respiratory disease transmission. Our results shed new light on nonviral respiratory illnesses in Sub-Saharan Africa.

## 2 Methods

### 2.1 Study site, subjects, and sample collection

The design, methods, and study population for this study have been previously reported in detail (9). Briefly, we conducted a prospective cohort study between May 2019 and August 2021 in rural Western Uganda. Household characteristics relevant for respiratory disease risk in this region include 7.4% adult tobacco smoking prevalence (28), 89% use of solid biomass cooking fuel (charcoal) (29), 44% living in dwellings made with permanent wall materials (30), and 40% access to adequate indoor ventilation (29). 69% of the local population rely on subsistence farming for their livelihoods (30). After obtaining written informed consent from adult participants and parents of child participants as well as assent from children >8 years old, we enrolled 234 children (ages 3–11) and 30 adults (ages 22–51), some of whom were parents of the child participants. Each month, trained nurses collected monthly nasopharyngeal swabs and respiratory symptoms scores (9) from all participants at their schools, homes, or workplaces. After Uganda instated national lockdown for COVID-19 on March 20, 2020, we obtained permission from study participants and the Ugandan government to continue sampling adult participants and their children (*n* = 31) at their homes with strict biosafety precautions to protect participants and study team personnel. Although primary schools in Uganda did not reopen until January 2022, some lockdown restrictions began to loosen in October 2020 (e.g., opening businesses, major roads, and the international airport), a period we denote “late lockdown.” De-identification of participant data required for institutional ethics approval precluded collecting demographic data beyond age ranges for each cohort.

### 2.2 Viral diagnostics

We tested nasopharyngeal swabs using the NxTAG Respiratory Pathogen Panel (RPP) (Luminex Corporation, Austin, TX, USA) as previously described (31, 32). Immediately upon sample collection, Dacron swabs were placed in RNAlater preservation buffer (Thermo Fisher Scientific, Waltham, MA, USA) and stored at −20°C until shipment on dry ice to Madison, Wisconsin, facilitating molecular analysis. Nucleic acids were extracted as previously described using the NucliSENS EasyMag kit (bioMérieux, Marcy-l'Étoile, France) (31). The RPP tests for influenza viruses A and B, rhinovirus/enterovirus, adenovirus, parainfluenza viruses 1–4, coronaviruses (CoV NL63, CoV 229E, CoV HKU1, CoV OC43, and SARS-CoV-2), respiratory syncytial viruses A and B, metapneumovirus, human bocavirus, and the bacterial targets *Chlamydophila pneumoniae*, *Mycoplasma pneumoniae*, and *Legionella pneumophilia*. Sensitivity and specificity vary by pathogen but on average are approximately 95% and 99%, respectively (33).

### 2.3 Quantification of CXCL10 and respiratory bacteria

We measured CXCL10 mRNA levels via quantitative PCR (qPCR) in a representative subset of swabs (*n* = 232) to interrogate potential cryptic viral infections in samples that tested PCR-negative as described elsewhere (15) using specific primers (Table 1). Levels of *H. influenzae, M. catarrhalis,* and *S. pneumoniae* were measured in a subset of PCR-negative swabs from adults (*n* = 30) and children aged 3–6 years (*n* = 125) as described elsewhere (34–36) using specific primers (Table 1). CXCL10 and bacteria qPCR reactions were performed in 25 µl volumes consisting of 13.8 µl POWER SYBR Green PCR Master Mix (Thermo Fisher Scientific), 100 µM each primer, PCR-grade water, and 2 µl cDNA. Thermal cycling parameters consisted of an initial incubation of 50°C for 2 min and 95°C for 10 min, followed by 40 cycles of 95°C for 15 s and 60°C for 1 min. The qPCR assay was performed using a CFX96 Touch Real-Time PCR Detection System (Bio-Rad, Hercules, CA, USA).

**Table 1:** Primers used in this study.

Name	Sequence 5’ → 3’	Target gene
CXCL10-F	GCCATTCTGATTTGCTGCCT	CXCL10
CXCL10-R	GCAGGTACAGCGTACAGTTC	CXCL10
hpdF822	GGTTAAATATGCCGATGGTGTTG	*Haemophilus influenzae* hpd
hpdR952	TGCATCTTTACGCACGGTGTA	*Haemophilus influenzae* hpd
copB-F	GTGAGTGCCGCTTTACAACC	*Moraxella catarrhalis* copB
copB-R	TGTATCGCCTGCCAAGACAA	*Moraxella catarrhalis* copB
lytA-F	ACGCAATCTAGCAGATGAAGCA	*Streptococcus pneumoniae* lytA
lytA-R	TCGTGCGTTTTAATTCCAGCT	*Streptococcus pneumoniae* lytA

### 2.4 Metagenomic sequencing and bioinformatics

Metagenomic sequencing was used to identify viruses in 24 nasopharyngeal swabs from children who displayed moderate to severe respiratory symptoms (symptoms scores = 6–16) but tested virus-negative by RPP, using previously described methods (37–40). Briefly, Dacron swab tips were homogenized with 50 μl RNAlater suspension and 350 μl Hanks' Balanced Salt Solution (HBSS) and centrifuged to clarify. The supernatant was treated with nucleases to digest nucleic acids not encapsidated within virions (41). Nucleic acids were extracted using the QIamp MinElute Virus Spin Kit (Qiagen, Hilden, Germany), with carrier RNA omitted. RNA was converted to double-stranded cDNA with the Superscript IV system (Thermo Fisher), which was purified using Agencourt AMPure XP beads (Beckman Coulter, Brea, CA, USA) as previously described (37–40). Genomic libraries were prepared using the Illumina Nextera XT kit (Illumina, San Diego, CA, USA) and sequenced on an Illumina MiSeq instrument using 300 × 300 cycle paired-end (V3) chemistry.

Sequences of low quality (Phred score <30) and short length (<50 bp) were trimmed and sequences matching known contaminants and host DNA were discarded using CLC Genomics Workbench v. 20.0.4 (Qiagen, Hilden, Germany). Remaining reads were then subjected to *de novo* assembly using the metaviral option in SPAdes v. 3.15.2 (42). The resultant contiguous sequences (contigs) were compared to viruses in NCBI databases at both the nucleotide and amino acid levels using the BLASTn and BLASTx algorithms, respectively (43, 44).

### 2.5 Comparison of nonviral colds and bacteria colonization with suburban US children

Symptoms status, viral infection status, and bacteria colonization status were compared to data collected as part of the 2006–2008 RhinoGen study described in detail elsewhere (10, 22, 45). Three hundred eight children (166 with asthma and 142 without asthma) aged 4–12 years living in Madison, Wisconsin, a suburban college town in the US, were enrolled in the study. Children provided weekly nasal lavage samples (45), which have been shown to yield similar rates of bacterial detection as nasopharyngeal swabs (22). Methods utilized for viral diagnostics and qPCR for *H. influenzae, M. catarrhalis,* and *S. pneumoniae* were identical to those used in this study (10), allowing for direct comparison. Only age-matched children (4–6 years) were included (*n* = 289).

### 2.6 Statistical analysis

Parametric model assumptions were assessed with Shapiro-Wilk tests for verification of normality and with Levene's test for verification of homogeneity of variances. For power analysis for the bacteria assays, we utilized the functions *cohensD* and *pwr.t.test* in R (46) on a pilot subset of samples (*n* = 31), aiming for a significance level of 0.05 and a power of 0.8. Symptoms status, viral infection, and age class were compared to bacteria colonization by *X*^2^ test or Fisher's exact test for association. Presence of nonviral colds or bacteria colonization were compared to calendar month with ANOVA or Kruskal-Wallis test. Levels of CXCL10 or bacteria, measured by qPCR Ct values, were compared to age class or symptoms status by Mann-Whitney *U* test. A two-sided *p*-value of less than 0.05 was regarded as statistically significant.

## 3 Results

In total, we collected 2,047 symptoms scores (534 scores from 30 adults and 1,513 scores from 234 children) and 1,989 nasopharyngeal swabs from 264 individuals (538 swabs from adults and 1,451 swabs from children) from May 2019 through August 2021. We obtained 1,976 paired swabs and symptoms scores.

### 3.1 Nonviral colds

**Figure 1:**
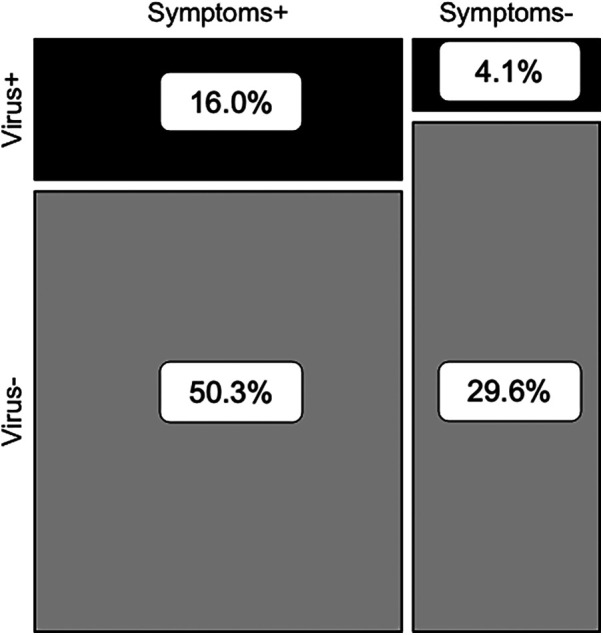
Contingency analysis of presence/absence of viral respiratory pathogens and respiratory symptoms (*n* = 1,976 paired nasopharyngeal swabs and symptoms scores).

**Figure 2:**
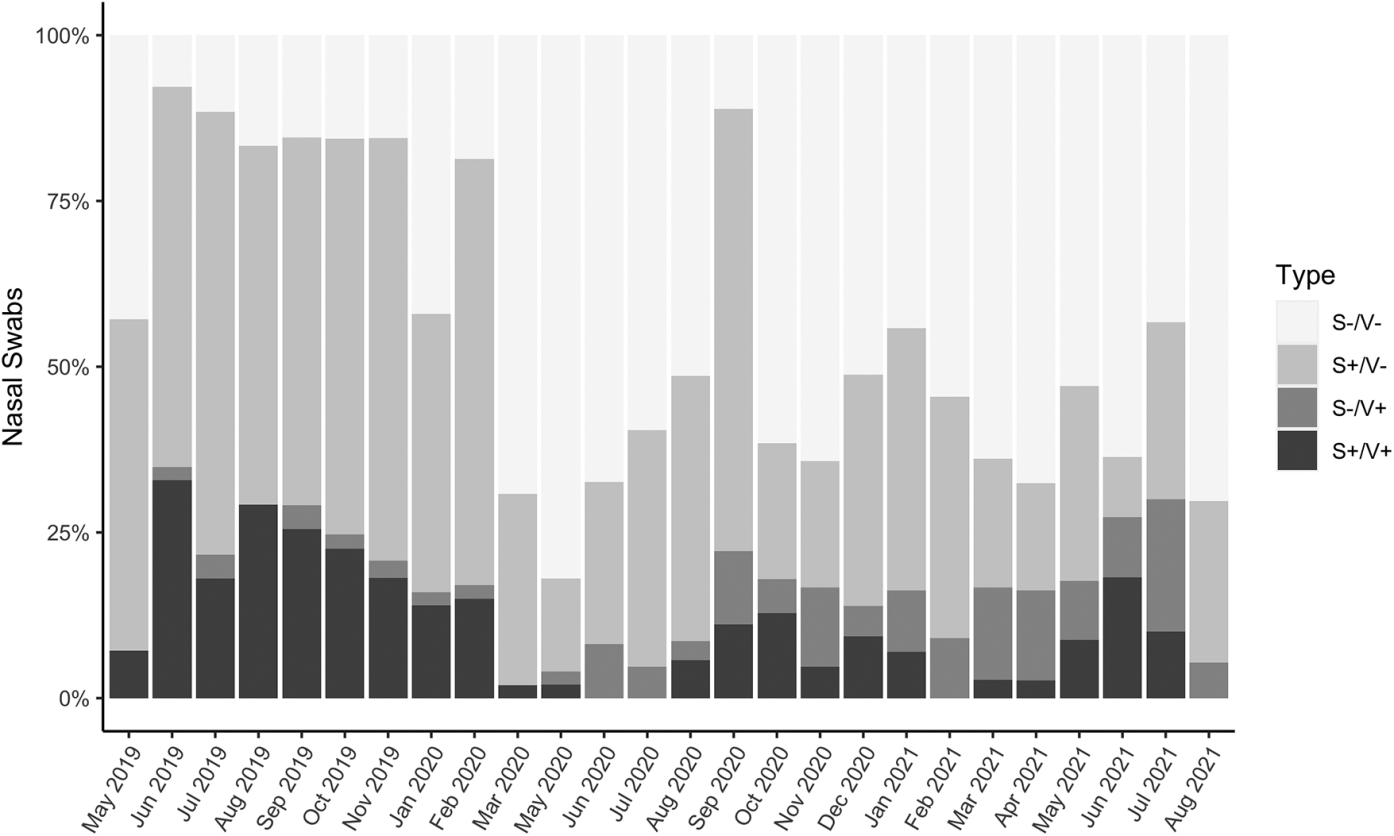
Proportions of symptoms-negative/virus-negative (S-/V-; healthy), symptoms-positive/virus-negative (S+/V-; nonviral cold), symptoms-negative/virus-positive (S-/V+; asymptomatic infection), and symptoms-positive/virus-positive (S+/V+; symptomatic infection) samples, May 2019-August 2021 (*n* = 1,976 paired nasopharyngeal swabs and symptoms scores). There were no samplings in December 2019 or April 2020.

If participants reported experiencing any respiratory symptoms, their nasopharyngeal swabs were 2.3 times more likely to test PCR-positive for at least one of 17 common respiratory viruses than if they experienced no symptoms (Fisher's exact test, *p* < 0.001) (Figure 1). However, samples which tested PCR-negative but were collected from people experiencing respiratory symptoms (“S+/V-”; *n* = 993) accounted for 50.3% of the total sample set (*n* = 1,976 paired swabs with symptoms scores) and 75.8% of all swabs collected during symptomatic disease (*n* = 1,310) (Figure 1). The proportion of S+/V- swabs decreased significantly during the most stringent period of lockdown (March 2020–September 2020; 6.0%) compared to the pre-pandemic (May 2019–February 2020; 23.6%) or late lockdown (October 2020–August 2021; 18.7%) periods (one-way ANOVA with Tukey HSD, pairwise *p* = 0.004 pre-pandemic vs. March–September 2020, pairwise *p* = 0.03 March–September 2020 vs. October 2020–August 2021) (Figure 2).

### 3.2 CXCL10 expression

**Figure 3:**
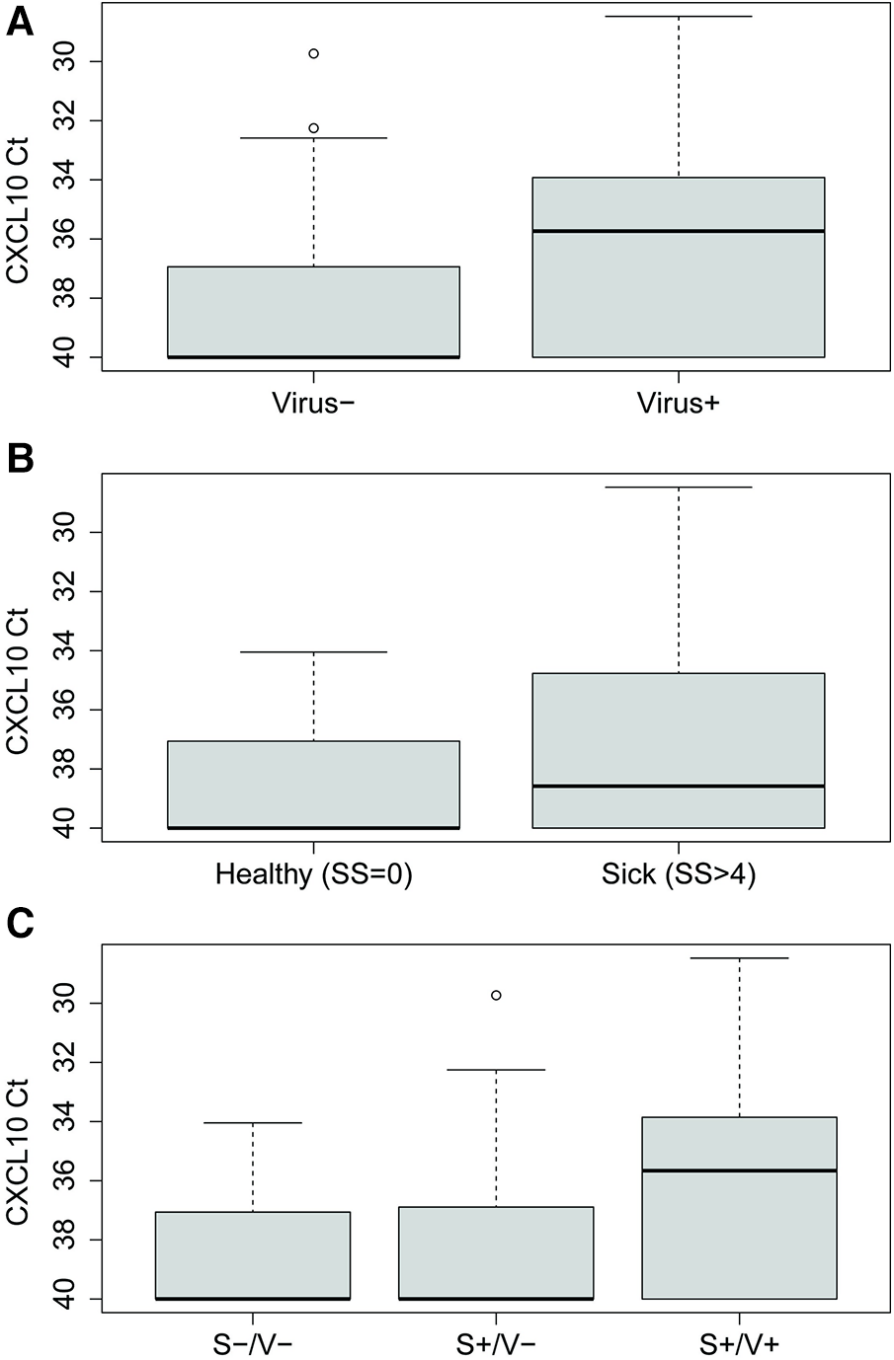
CXCL10 expression levels quantified by Ct values for (**A**) virus-positive (*n* = 100) vs. virus-negative (*n* = 132) people, as diagnosed by multiplex PCR panel, (**B**) no symptoms (*n* = 23) vs. moderate-to-severe symptoms (*n* = 209), as measured by symptoms scores (SS), and (**C**) symptoms-negative/virus-negative (S-/V-) (*n* = 23), symptoms-positive/virus-negative (S+/V-) (*n* = 109), and symptoms-positive/virus-positive (S+/V+) (*n* = 100) people.

CXCL10 expression levels were higher in virus-infected people than virus-negative, as determined by multiplex PCR (Mann-Whitney *U* test, *p* < 0.0001) (Figure 3A). People experiencing moderate to severe cold symptoms (symptoms scores > 4), regardless of etiology, had higher CXCL10 levels than people experiencing no cold symptoms (symptoms scores = 0) (Mann-Whitney *U* test, *p* = 0.032) (Figure 3B). Experiencing cold symptoms with a PCR-positive viral infection (S+/V+) was associated with increased CXCL10 levels compared to S+/V- cases (Kruskal-Wallis test with Dunn's multiple comparison, *p* < 0.0001) and S-/V- cases (Kruskal-Wallis test with Dunn's multiple comparison, *p* = 0.0001) (Figure 3C).

### 3.3 Metagenomic sequencing

Following quality trimming and in silico subtraction of host and known contaminant sequences from the sequenced S+/V- samples, we retained a total of 26,756,296 reads with an average length of 141.5 bp for analysis. No reads mapped to genomes of mammalian viruses, whereas 100% of reads matched phage, bacteria, or fungi (data not shown).

### 3.4 Bacterial detection in children and adults

**Figure 4:**
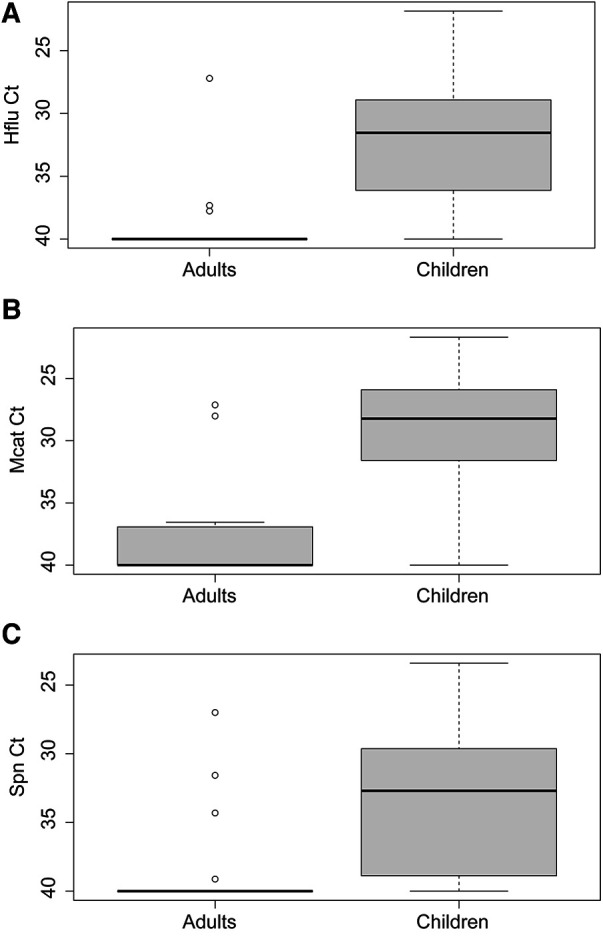
Levels of (**A**) *Haemophilus influenzae* (Hflu), (**B**) *Moraxella catarrhalis* (Mcat)*,* and (**C**) *Streptococcus pneumoniae* (Spn) in nasopharyngeal swabs of virus-negative adults (*n* = 30) and children (*n* = 125).

Prevalence of respiratory pathobionts *H. influenzae*, *M. catarrhalis,* and *S. pneumoniae* detected in nasopharyngeal swabs was higher in children than adults (87.2% vs. 20.0%, *X*^2 ^= 167.7, *p* < 0.0001) (Table 2). Levels were also significantly higher in children than adults for each bacterial species (Mann-Whitney *U* test, *p* < 0.0001 for each species) (Figure 4). Of the three species, *M. catarrhalis* was detected with the highest frequencies (*X*^2 ^= 16.6, *p* = 0.0002) (Table 2) and levels (Kruskal-Wallis with Dunn's multiple comparison, *p* < 0.0001) (Figure 4).

**Table 2:** Prevalence of respiratory pathobionts *Haemophilus influenzae*, *Moraxella catarrhalis,* and *Streptococcus pneumoniae* detected in nasopharyngeal swabs of virus-negative adults (*n* = 30) and children (*n* = 125).

Bacteria	Adults *n* (%)	Children *n* (%)
*Haemophilus influenzae*	30 (10.0)	109 (87.2)
*Moraxella catarrhalis*	11 (36.7)	121 (96.8)
*Streptococcus pneumoniae*	4 (13.3)	97 (77.6)

Totals do not add up to sample sizes due to coinfection in some individuals (see Table 3).

Coinfection was much more likely in children than in adults (Table 3). Coinfection was also more common than monoinfection in children, whereas the opposite was true for adults. The most common colonization status in children was infection with all three pathobionts. No children tested negative for all three pathobionts, whereas a triple-negative was the most common colonization status in adults.

**Table 3:** Respiratory pathobiont coinfection in adults (*n* = 30) and children (*n* = 125).

Bacteria	Adults *n* (%)	Children *n* (%)
*Haemophilus influenzae* only	1 (3.3)	2 (1.6)
*Moraxella catarrhalis* only	9 (30.0)	8 (6.4)
*Streptococcus pneumoniae* only	2 (6.7)	1 (0.8)
Hflu + Mcat	0 (0.0)	18 (14.4)
Hflu + Spn	0 (0.0)	1 (0.8)
Mcat + Spn	0 (0.0)	7 (5.6)
Hflu + Mcat + Spn	2 (6.7)	88 (70.4)
None	16 (53.3)	0 (0.0)

Hflu = *Haemophilus influenzae*; Mcat = *Moraxella catarrhalis*; Spn = *Streptococcus pneumoniae*.

Colonization with any of the three bacterial species was not associated with increased symptoms scores in either children or adults (Supplementary Table S1). Positive cases also were not associated with sampling month for any of the three bacteria (Kruskal-Wallis test, all pairwise comparisons *p* > 0.05) (Supplementary Figure S1).

### 3.5 Comparison of nonviral cold prevalence and bacteria colonization with US suburban children

Age-matched children living in a suburban US community (see Methods) were just as likely as rural Ugandan children from this study to have PCR-positive viral infections when they experienced respiratory symptoms (OR: 2.2 vs. 2.3; see Figure 1). However, the Ugandan children had frequencies of nonviral colds two times higher than the US children (50.2% vs. 25.3% of samples). Prevalence of all three bacterial species was significantly higher in the Ugandan children (Hflu: *X*^2^ = 151.0, *p* < 0.0001; Mcat: *X*^2^ = 154.2, *p* < 0.0001; Spn: *X*^2^ = 17.4, *p* < 0.0001) than in the virus-negative US children (Table 4). While nearly a third (32.9%) of the virus-negative US children were PCR-negative for all three pathobionts, no Ugandan children similarly tested triple-negative (Table 5). If the US children tested positive for any bacteria, the most commonly detected was *S. pneumoniae* (25.3%), whereas *S. pneumoniae* only was the least common detection (0.8%) in the Ugandan children (Table 5).

**Table 4:** Prevalence of respiratory pathobionts *Haemophilus influenzae*, *Moraxella catarrhalis,* and *Streptococcus pneumoniae* detected in nasal samples of age-matched, virus-negative Ugandan children (*n* = 125) and US children (*n* = 289).

Bacteria	Ugandan children^a^ *n* (%)	US children *n* (%)
*Haemophilus influenzae*	109 (87.2)	63 (21.8)
*Moraxella catarrhalis*	121 (96.8)	86 (29.8)
*Streptococcus pneumoniae*	97 (77.6)	160 (55.4)

^a^ Values identical to Table 2.

**Table 5:** Respiratory pathobiont coinfection in nasal samples of age-matched, virus-negative Ugandan children (*n* = 125) and US children (*n* = 289).

Bacteria	Ugandan children^a^ *n* (%)	US children *n* (%)
*Haemophilus influenzae* only	2 (1.6)	13 (4.5)
*Moraxella catarrhalis* only	8 (6.4)	19 (6.6)
*Streptococcus pneumoniae* only	1 (0.8)	73 (25.3)
Hflu + Mcat	18 (14.4)	2 (0.7)
Hflu + Spn	1 (0.8)	22 (7.6)
Mcat + Spn	7 (5.6)	39 (13.5)
Hflu + Mcat + Spn	88 (70.4)	26 (9.0)
None	0 (0.0)	95 (32.9)

Hflu = *Haemophilus influenzae*; Mcat = *Moraxella catarrhalis*; Spn = *Streptococcus pneumoniae*.

^a^ Values identical to Table 3.

## 4 Discussion

In this study of respiratory illness in rural Western Uganda, people were 2.3 times more likely to test PCR-positive for at least one of 17 common respiratory viruses if they were experiencing respiratory symptoms than if they felt healthy. However, 50.2% of all nasopharyngeal swabs, including 75.8% of swabs collected during symptomatic disease, tested PCR-negative for respiratory viruses (S+/V-), which was confirmed by metagenomic sequencing for a subset of samples with high symptoms scores. We then tested S+/V- samples for the respiratory pathobionts *H. influenzae*, *M. catarrhalis,* and *S. pneumoniae* and found that all child participants tested positive for at least one bacterial species, with coinfections of all three species as the most common presentation (70.4% of swabs). These children had much higher prevalence rates and levels of these bacteria than adults as well as much higher prevalence rates compared to age-matched children living in the suburban US community of Madison, Wisconsin. Adults tested positive for each of the three pathobionts, although detection rates in adults were much lower than in children.

The prevalence of nonviral colds was much higher in our study (50.2%) in rural Western Uganda than recorded elsewhere in the world. Nonviral colds accounted for 22% of samplings in the Tecumseh study, a seminal respiratory disease cohort study performed in the US in the 1960s (2, 47), as well as 31% of samplings in Finland in the 1990s (4). Despite using the same diagnostic methods as in this study, nonviral colds only accounted for 25.3% of samplings among age-matched children in Madison, Wisconsin, in the RhinoGen study (10). It has been argued that purportedly nonviral colds may be caused by viruses yet to be identified (48), especially after the discoveries of human metapneumovirus (49) and human bocavirus (50) in the past 20 years. However, we did not find any novel viruses in these samples using metagenomic DNA sequencing, a technique our group has used extensively to identify novel infectious agents in a variety of host species (38, 51, 52).

On average, S+/V- samples had lower CXCL10 expression than S+/V+ samples, concurring with current understanding that CXCL10 expression is increased significantly during inflammation induced by viral infection (11), including by respiratory viruses such as rhinovirus (53), respiratory syncytial virus (54), and coronaviruses (55). However, CXCL10 expression is not always specific to viral infection, as evidenced by the S+/V- samples that also had higher CXCL10 expression than S-/V- samples, despite testing negative for viruses by multiplex PCR panel and metagenomic sequencing. Indeed, CXCL10 expression has been demonstrated to increase in response to infection with bacterial and protozoal infections common in Uganda, such as tuberculosis (56), scrub typhus (57), malaria (58), and leishmaniasis (59), as well as in noninfectious inflammatory disorders, such as asthma (60) and chronic obstructive pulmonary disorder (COPD) (61). We therefore explored nonviral causes of S+/V- illnesses in our data set.

Carriage of respiratory pathobionts *H. influenzae*, *M. catarrhalis,* and *S. pneumoniae* was ubiquitous among young children experiencing nonviral colds in our study; 100.0% of S+/V- swabs from children tested positive for at least one species. Although the goal of the current study was to elucidate causes of nonviral colds, previous studies have shown that pathobiont carriage increases with respiratory viral infections (10, 22) and data for this population in future studies would be a valuable comparison. For each bacterial species, detection rates were much higher than those among children in Madison, Wisconsin, using the same qPCR methods as this study (10). There is growing evidence that pathobiont carriage rates among children differ geographically (62) and by socioeconomic status (63). For example, rates were similar to those measures elsewhere in Sub-Saharan Africa among Gambian neonates (64) as well as HIV-positive children living in Tanzania (65) and Ethiopia (66). Seasonality did not appear to affect pathobiont carriage, as has been reported in other studies (67–69), in which rates were inversely proportional to temperature with a peak during winter months. However, these studies all took place in temperate regions of the US where seasonality is more pronounced. It is possible that patterns of respiratory pathobiont colonization are more variable in tropical regions, similar to patterns described for respiratory viruses (70, 71).

Pathobiont colonization was not associated with increased symptoms severity, in contrast to previous studies (16, 22). Because children in this study reported experiencing respiratory symptoms during 80.3% of samplings and adults reported symptoms only 26.6% of the time (9), it is possible we did not have the statistical power to differentiate symptoms status by colonization status. In fact, we assayed every sample from children with no symptoms and adults with at least moderate symptoms (symptoms scores > 4) and we still failed to detect significant differences between these groups and the more common symptomatic children and asymptomatic adults, respectively. Mechanisms explaining the observed association between pathobiont colonization and respiratory symptom severity in our study cohorts therefore remain unknown. Nonetheless, high pathobiont detection rates in early childhood have well known associations with clinically significant conditions, such as acute otitis media, sinusitis, pneumonia, and asthma (16). Children in this population may be at increased risk for these conditions. Future studies should investigate pathobiont diversity measures (e.g., alpha and beta diversity) and Th1 cytokine pathways beyond CXCL10 to further characterize the relationship between colonization with these bacteria and airway inflammation.

Although adults had much lower frequencies of respiratory pathobiont colonization than children, these frequencies, especially for *M. catarrhalis*, were still higher than those recorded elsewhere, including among healthy adults in England (72) and adults with COPD in the US (17). In fact, *M. catarrhalis, H. influenzae,* and *S. pneumoniae* cause approximately half of COPD exacerbations among adults (18). Chronic bacterial colonization in the respiratory tract leads to sloughing of highly immunogenic cell wall antigens that leads to the hallmark airway inflammation of COPD (18). COPD is a growing problem across Sub-Saharan Africa and is expected to overtake HIV/AIDS as the leading cause of death in this region by 2030 (73). In previous study in Uganda, place of residence (rural vs. urban) was the most significant determinant of COPD diagnosis, with COPD being more prevalent in rural areas (74). Asymptomatic carriage of these bacteria, however, is common among adults (18) and children (75, 76). Thus, PCR positivity should not be used exclusively to diagnose COPD in study participants, although we suspect it may contribute to the high prevalence of nonviral respiratory illnesses.

Other potential causes of nonviral colds in children and adults include allergic rhinitis, asthma, or air pollutant exposure. Respiratory allergies can also provoke non-infectious nasal and chest symptoms in children, but is an unlikely explanation for symptoms in Uganda, where the prevalence of allergic rhinitis is <5% (77). Evidence from other studies suggests that asthma may be underdiagnosed in Uganda, both in young children (78) and adults (79). Critically, however, both indoor and outdoor air pollution in Western Uganda regularly exceed levels deemed unsafe by the WHO by four to six times (80). Annual mean PM_2.5_ concentrations in the Western Uganda region measure in the top quintile for the country (27). Smoke and carbon monoxide from indoor charcoal-fueled cookstoves used in the area may contribute to respiratory disease symptoms (29), including asthma in children (81) and COPD exacerbations in adults (73). This association is not unique to Uganda or Sub-Saharan Africa. In urban areas of the US, spikes in air pollutants similarly can produce upper and lower respiratory illnesses in children (82). Air pollution also exacerbates COPD in adults all over the world (83). We advocate for mitigating exposure to non-infectious respiratory disease factors that are prevalent in rural Uganda, such as indoor biomass smoke and outdoor air pollution from vehicles, industrial and agricultural practices, or fires (84).

In summary, our findings demonstrate that people living in rural Western Uganda experience high frequencies of nonviral colds. Although we detected high detection rates of respiratory bacteria, especially in children, carriage was not associated with increased cold symptoms severity. However, this may be due to the low number of children not reporting any respiratory symptoms in the study. Our data do not support treatment of these bacteria as a way to reduce the frequency and severity of nonviral colds in this population. Although there is evidence that azithromycin reduces wheezing illness in babies and preschool-aged children (85, 86), suggesting a relationship between wheezing and airway bacteria dysbiosis (87), serious risks include antimicrobial resistance, killing healthy microbes, drug costs, and potential side effects. Antibiotic therapy therefore is not advised in uncomplicated colds.

Vaccinations against these pathobionts are either already in use for *S. pneumoniae* (PCV13) (88) or in development for *M. catarrhalis* and non-typeable *H. influenzae* (89). Since they were first licensed in 2000, pneumococcal conjugate vaccines have altered population-level detection rates of non-vaccine type *S. pneumoniae* (90)*, H. influenzae* (91, 92)*,* and *M. catarrhalis* (91)*.* Although the current study is limited by a lack of individual participant health and demographic information, such as age and immunization status, due to required data de-identification, differential immunization rates between the US and Uganda could explain the differences in pathobiont colonization we observed between the two populations compared here. Indeed, childhood immunization rates are currently “suboptimal” in Uganda (93). For example, Uganda introduced PCV10 in 2013, later than neighboring Kenya (2011), Tanzania (2013) or Rwanda (2010), after experiencing funding challenges that hindered vaccine rollout and health worker training (94, 95). In July 2023, the official Ugandan government estimate for full three-dose PCV coverage was 90% [at the time of the RhinoGen study, PCV coverage in the US was 93% after introduction in 2000 (96)], but the Ugandan figure was disputed by the WHO and UNICEF estimates of national immunization coverage (WUENIC) because no nationally representative household survey had been conducted in the previous five years (97). Closing this gap in vaccine uptake presents an opportunity for increasing protection against respiratory illnesses and their sequelae in Ugandan children.

Incidentally, our study occurred before, during, and after the emergence of COVID-19 in Uganda. We documented that the proportion of S+/V- samples decreased sharply during the most stringent part of COVID-19 lockdown in Uganda from March to September 2020, mirroring a drop in respiratory viral illnesses observed around the world during the same period (98). The decrease in nonviral colds we observed may have been associated with nonpharmaceutical interventions relied upon before the introduction of COVID-19 vaccines that would have protected mask-wearers from inhaling noxious particulate matter (99) and children staying home from school from pathobiont transmission (100). Lockdown had many deleterious social and economic effects across Sub-Saharan Africa due to lost social safety nets, daily wages, and educational opportunities (101–103). However, our findings support the fact that there were significant improvements to respiratory health in rural Uganda, particularly among children. Therefore, future public health policy measures should build upon these gains by exploring strategies that are more sustainable in the long-term. For example, based on the results of this study, the local government health office has changed its clinical response to respiratory disease in our study population by reducing overcrowding in primary school classrooms and laying cement over dirt floors to avoid particulate inhalation. We hope this study continues to serve as a model of how such research can have clinical implications and result in direct, meaningful, and specific changes in practice.

## Data Availability

The datasets presented in this article are not readily available because of research ethics requirements to protect study participants' health information privacy. Requests to access the datasets should be directed to the corresponding author (tony.goldberg@wisc.edu).
