# Supplementary material for: High frequencies of nonviral colds and respiratory bacteria colonization among children in rural Western Uganda
Source: Front Pediatr. 2024 May 2;12:1379131. doi: 10.3389/fped.2024.1379131 (PMC11096560; doi:10.3389/fped.2024.1379131)
Supplement: Supplementary file 1 [file Datasheet1.docx]

Supplementary Material

# Supplementary Tables

**Supplementary Table 1.** Presence of moderate to severe symptoms (symptoms score>4) and respiratory bacteria colonization.

| Bacteria | Adults  (X^2^) | Children  (X^2^) |
| --- | --- | --- |
| *Haemophilus influenzae* | 0.01, p=0.9029 | 0.93, p=0.3343 |
| *Moraxella catarrhalis* | 0.00, p=1.0000 | 0.48, p=0.4903 |
| *Streptococcus pneumoniae* | 0.46, p=0.4953 | 0.95, p=0.3286 |
| Any pathobiont | 0.34, p=0.5612 | 1.11, p=0.2931 |

# Supplementary Figures


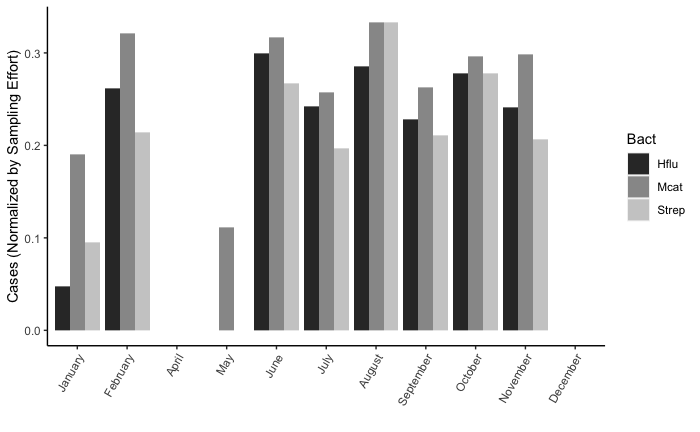


**Supplementary Figure 1.** Month of sampling and respiratory bacterial colonization. Positive cases were normalized by number of swabs tested in each month. No samplings were performed in March, and only adults were sampled in April or December. Hflu=*Haemophilus influenzae*; Mcat=*Moraxella catarrhalis*; Strep=*Streptococcus pneumoniae*.
